# Extranodal presentation of a lymphoma with precursor B-cell phenotype and translocation t(8;14) in South Africa

**DOI:** 10.4102/ajlm.v11i1.1355

**Published:** 2022-01-31

**Authors:** Katherine E. Hodkinson, Yvonne Perner, Deborah K. Glencross, Tracey Wiggill, Adam Botha, Janet Poole

**Affiliations:** 1 Department of Molecular Medicine and Haematology, University of the Witwatersrand, Johannesburg, South Africa; 2 National Health Laboratory Services, Johannesburg, South Africa; 3 Department of Anatomical Pathology, University of the Witwatersrand, Johannesburg, South Africa; 4 Department of Haematology and Molecular Medicine, University of the Witwatersrand, Johannesburg, South Africa; 5 Department of Paediatric Oncology, University of the Witwatersrand, Johannesburg, South Africa; 6 Department of Paediatric Oncology, Charlotte Maxeke Johannesburg Academic Hospital, Johannesburg, South Africa

**Keywords:** Burkitt leukaemia/lymphoma, B-lymphoblastic leukaemia/lymphoma, translocation t(8;14), variant MYC translocations, S-phase fraction, terminal deoxynucleotidyl transferase (TdT), extranodal

## Abstract

**Introduction:**

A rare entity of a B-cell malignancy with precursor B-cell phenotype and concomitant translocation t(8;14) or variant MYC translocation exists. These cases show clinical, pathological and molecular overlap between precursor B-lymphoblastic leukaemia or lymphoma and Burkitt leukaemia or lymphoma (BLL).

**Case presentation:**

We report a case from February 2019 at the Charlotte Maxeke Johannesburg Academic Hospital, South Africa, of a 9-month-old infant with a predominantly extracranial soft tissue mass showing extradural extension. There was no involvement of the peripheral blood or bone marrow. Fine needle aspiration and Tru-Cut biopsy of the soft tissue scalp mass showed the tumour to be of precursor B-cell phenotype. Contrastingly, an immunophenotypic assessment revealed a high S-phase fraction and raised concern for BLL. This prompted testing for the translocation t(8;14) by fluorescence in-situ hybridisation analysis, which confirmed this aberration.

**Management and outcome:**

Based on the published experience of other centres, the patient was initiated on a BLL protocol. Despite an excellent clinical response, the patient succumbed to neutropenic sepsis six months after diagnosis.

**Conclusion:**

Leukaemia or lymphoma with translocation t(8;14) or variant MYC translocation and precursor B-cell phenotype is a rare entity with a varied clinical presentation. This poses a challenge for diagnosis and classification and a clinical dilemma for the choice of treatment.

## Introduction

The presence of the translocation t(8;14) or variant MYC translocation in leukaemia with a precursor B-cell phenotype has been described in approximately 2% of paediatric cases.^1,2^ Rarer still is a pure lymphomatous version (extranodal or nodal), which, to the best of our knowledge, has only been reported twice in the literature, one of which was in a paediatric patient.^3,4^

This entity has overlapping clinical and pathological features with Burkitt leukaemia or lymphoma (BLL) and precursor B-lymphoblastic leukaemia or lymphoma (B-ALL). It has been described across a spectrum of ages ranging from 32 months to 64 years, in both male and female patients. Peripheral blood and bone marrow involvement have been reported in the majority of cases, with isolated extranodal disease reported in only a few.^1,3,4,5^ The immunophenotype is that of a precursor B-cell, with the expression of CD19, CD10 and terminal deoxynucleotidyl transferase (TdT), varied expression of CD34, and no expression of both surface light chain and CD20. Molecular studies have confirmed the involvement of the MYC gene in a translocation t(8;14) or variant translocation, which is the molecular hallmark of BLL.^2^ This genetic aberration has largely directed the choice of therapy towards a mature B-cell or Burkitt-like protocol in almost all reported cases. It is uncertain whether this represents the best therapeutic approach as there is very limited data on both the duration of remission in these patients and the exact underlying molecular behaviour of this tumour.

This case study highlights this rare entity, as well as its associated diagnostic and therapeutic challenges.

## Ethical considerations

Parental informed consent was provided for this case report. The ethical clearance was obtained from the University of the Witwatersrand Human Research Ethics Committee (Medical), Johannesburg, South Africa, under the approval number M190356. Patient results were de-identified and stored in a secure database to ensure patient confidentiality.

## Case presentation

A 9-month-old male patient from Angola presented to the Charlotte Maxeke Johannesburg Academic Hospital, South Africa, in February 2019, with a 3–4-week history of progressive bilateral proptosis and scalp masses. The computed tomography scan identified bilateral occipital and temporal scalp masses with extracranial extension into the orbital cavities and sinuses, and extradural extension into the anterior cranial fossa. There was no hepatosplenomegaly or lymphadenopathy. A fine needle aspiration sample of the soft tissue scalp mass was submitted for flow cytometry, and a Tru-Cut biopsy (manufacturer unknown) for histological assessment at the National Health Laboratory Service. As part of the staging work up, bone marrow aspiration and a trephine biopsy were performed to exclude infiltration of the marrow.

### Laboratory methods

Flow cytometry was performed on the fine needle aspiration sample from the scalp mass on a FACS Calibur instrument (BD Biosciences, San Jose, California, United States), using the PAINT-A-GATE (Becton Dickinson, BD Biosciences, San Jose, California, United States) and MODFIT (Verity Software House, Topsham, Maine, United States) software. The following antibodies were used: CD19 FITC (Becton Dickinson, BD Biosciences, San Jose, California, United States), CD10 PE (Beckman Coulter Inc., Brea, California, United States), CD45 PERCP (Becton Dickinson, BD Biosciences, San Jose, California, United States), CD34 APC (Becton Dickinson, BD Biosciences, San Jose, California, United States), CD117 PE (Beckman Coulter Inc., Brea, California, United States), HLA-DR FITC (Becton Dickinson, BD Biosciences, San Jose, California, United States), CD13 PE (Beckman Coulter Inc., Brea, California, United States), Kappa FITC (Dako, Glostrup, Denmark), Lambda PE (Dako, Glostrup, Denmark) and cytoplasmic TdT FITC (Dako, Glostrup, Denmark).

Immunohistochemical work up was performed on the Tru-Cut biopsy from the scalp mass using the following stains: CD20 (Dako, Glostrup, Denmark), PAX5 (Dako, Glostrup, Denmark), CD10 (Leica Biosystems, Newcastle Upon Tyne, United Kingdom), TdT (Cell Marque, Rocklin, California, United States), CD34 (Dako, Glostrup, Denmark), EBER ISH (Roche, Mannheim, Germany), BCL2 (Dako, Glostrup, Denmark), and Ki67 (Dako, Glostrup, Denmark).

Fluorescence in-situ hybridisation for the detection of translocation t(8;14) was performed using a Vysis LSI MYC/IGH/CEP8 tri-colour dual fusion probe (Abbott, Chicago, Illinois, United States), with orange, green and aqua signals reflecting the MYC, IgH and centromere 8 regions.

### Laboratory results

**FIGURE 1:**
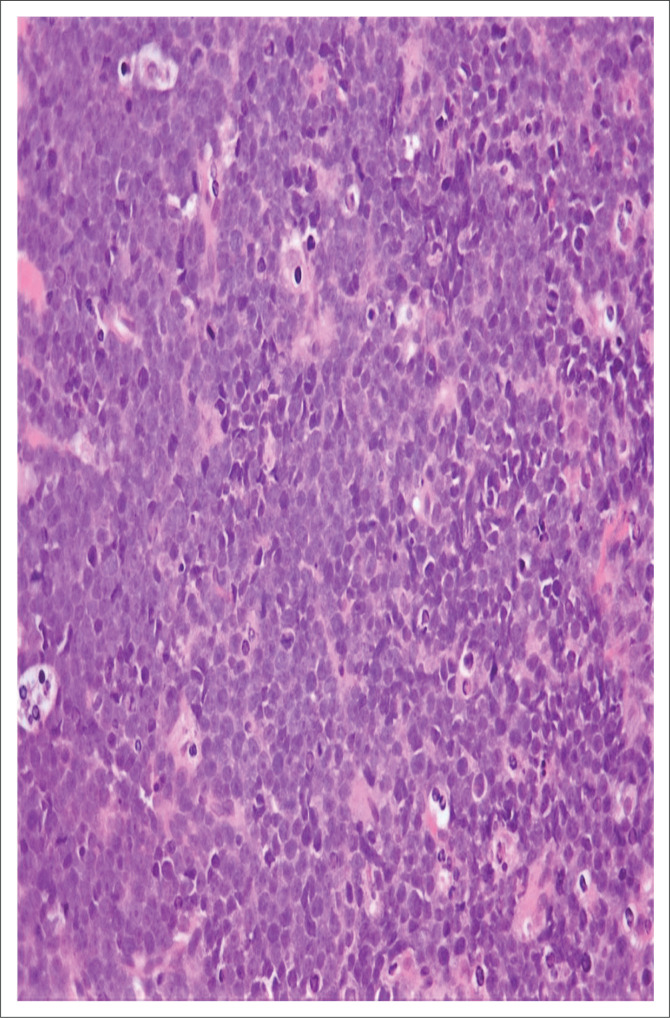
Tru-Cut biopsy of scalp mass of a 9-month-old male patient who presented to the Charlotte Maxeke Johannesburg Academic Hospital in February 2019. Image shows diffuse infiltration by intermediate-sized tumour cells with dispersed nuclear chromatin, irregular nuclear contours and one to four inconspicuous nucleoli. Numerous tingible body macrophages impart a starry sky pattern. 7 mitotic figures per 40 HPF. Haematoxylin & eosin (H&E) stain at ×40 magnification.

**FIGURE 2:**
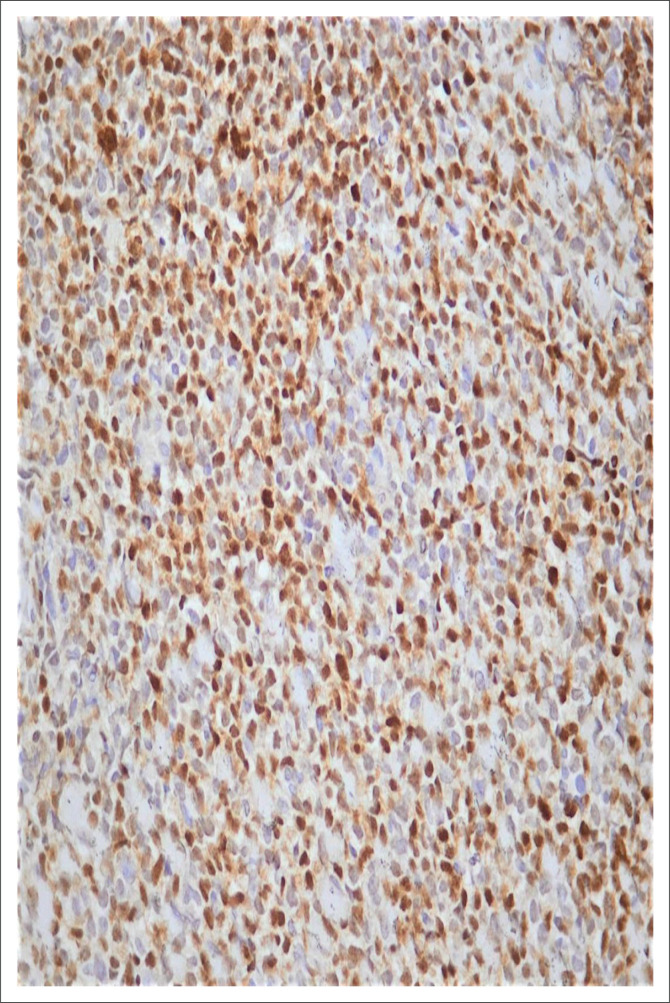
Terminal deoxynucleotidyl transferase (TdT) immunohistochemical stain of Tru-Cut biopsy of scalp mass from a 9-month-old male patient who presented to the Charlotte Maxeke Johannesburg Academic Hospital in February 2019. Image shows ~40% positivity in the tumour. ×40 magnification.

**FIGURE 3:**
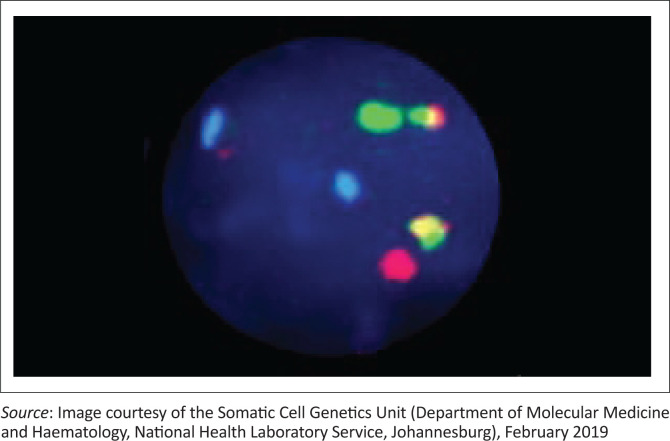
Fluorescence in-situ hybridisation analysis of fine needle aspirate from a 9-month-old male patient who presented to the Charlotte Maxeke Johannesburg Academic Hospital in February 2019. Image shows translocation t(8;14)(q24;q32) using the Vysis LSI MYC/IGH/CEP8 tri-colour dual fusion probe, as well as a 2F1O1G2A pattern: 2 fusion, 1 orange, 1 green, 2 aqua signals. The orange, green and aqua signals represent the MYC, IgH and centromere 8 regions.

Histological examination of the Tru-Cut biopsy of the scalp mass revealed intermediate-sized tumour cells with irregular nuclear contours, a high nuclear-cytoplasmic ratio, dispersed nuclear chromatin, and one to four inconspicuous nucleoli present within a prominent background of tingible body macrophages (Figure 1). The tumour immunophenotype based on flow cytometry and immunohistochemistry was that of a precursor B-cell, with the expression of TdT, absence of surface light chains and a high Ki67 index (Table 1, Table 2 and Figure 2). Contrastingly, the high S-phase fraction detected was in a range that is typically seen in Burkitt leukaemia.^6^ Given the latter, Fluorescence in-situ hybridisation analysis was requested and found to be positive for the translocation t(8;14)(q24;q32) (Figure 3).

**TABLE 1:** Laboratory results at presentation of a 9-month-old male patient who presented to the Charlotte Maxeke Johannesburg Academic Hospital in February 2019.

Laboratory tests	Results
**Blood results**	
White cell count (× 10^9^/L)	5.8
Absolute neutrophil count (× 10^9^/L)	2.95
Haemoglobin (g/dL)	7.5
Mean cell volume (f/L)	65.4
Mean cell haemoglobin concentration (g/dL)	29
Platelet count (× 10^9^/L)	734
LDH (U/L)	682
Ferritin (ug/L)	13
Uric acid (mmol/L)	0.22
Bone marrow infiltration (Based on morphology, IgH gene rearrangement studies, cytogenetic analysis)	Absent
Cerebrospinal fluid involvement (based on cytology, flow cytometry, IgH gene rearrangement studies)	Absent

IgH, Immunoglobulin heavy chain; LDH, Lactate dehydrogenase.

**TABLE 2:** Laboratory results at presentation of a 9-month-old male patient who presented to the Charlotte Maxeke Johannesburg Academic Hospital in February 2019.

Immunophenotype	FNA Flow cytometry	Tru-Cut biopsy IHC
CD19	+	NT
	(bright)	
CD20	NT	−
CD22	NT	NT
PAX5	NT	+
CD10	+	+
	(bright)	
Human leukocyte antigen – DR isotype	+	NT
CD45	+	NT
	(dimmer than the background lymphocytes)	
TdT	+ in ~30%	+ in ~30% – 40%
CD34	−	−
CD117	−	NT
CD13	dim +	NT
Light chain restriction	−	NT
S-phase fraction	35.7%	NT
Ki67	NT	~100%
BCL2	NT	+
EBER ISH	NT	−
*FISH analysis* t(8;14)(q24;q32)	+	+
IgH gene rearrangement studies	Monoclonal	NT

-, negative; +, positive; BCL2, B-cell lymphoma 2; EBER ISH, Epstein–Barr virus-encoded small RNA in-situ hybridisation; FISH, fluorescence *in-situ* hybridisation; FNA, fine needle aspirate; IgH, Immunoglobulin heavy chain; IHC, immunohistochemistry on the Tru-Cut biopsy of the scalp mass; LDH, Lactate dehydrogenase; NT, not tested; TdT, Terminal deoxynucleotidyl transferase.

## Management and outcomes

The patient was initiated on a BLL regimen as per the FAB LMB 95 protocol with excellent clinical response.^7^ There was complete resolution of the proptosis and the scalp masses were no longer clinically evident. Sadly, the patient succumbed to neutropenic sepsis six months after diagnosis.

## Discussion

The co-existence of the translocation t(8;14) or variant MYC translocation with a precursor B-cell phenotype in an extranodal malignancy is extremely uncommon and has been reported in only one paediatric and one adult patient in the literature.^3,4^ These individuals were both female and had no laboratory evidence of either peripheral blood or bone marrow involvement at presentation. The youngest, a 5-year-old, presented with abdominal, orbital and mandibular masses.^4^ The second, a 64-year-old, had multiple extranodal lesions and a chronic disease course.^3^ Similarly, the clinical presentation of our patient was with isolated extranodal disease. This pattern of involvement is often seen in Burkitt lymphoma and thus formed part of the differential diagnosis in our patient.

**FIGURE 4:**
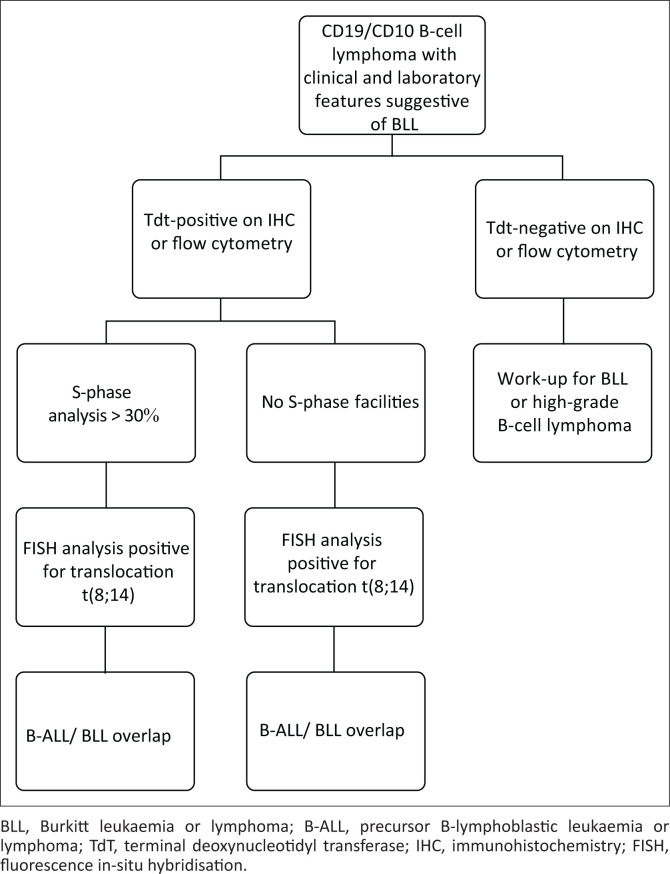
Laboratory approach to the diagnosis of lymphoma with features of precursor B-lymphoblastic leukaemia or lymphoma and Burkitt leukaemia or lymphoma.

Laboratory work up demonstrated the co-expression of CD19 and CD10 by the tumour, with no expression of CD34 or light chains. Because of this, the expression of TdT was assessed. Terminal deoxynucleotidyl transferase is a DNA polymerase that functions to provide junctional diversity in both B-cell and T-cell receptor genes at the precursor cell stage.^8^ It is a marker of immaturity and its expression in mature neoplasms such as BLL would be aberrant. Terminal deoxynucleotidyl transferase was found to be positive in ~30% – 40% of the tumour on both the fine needle aspiration and Tru-Cut biopsy specimens. While these results pointed towards a tumour at a precursor cell stage of development, the S-phase fraction analysis found the tumour proliferative activity to be very high. This measurement is performed by flow cytometry and uses DNA content to determine the proportion of cells in each phase of the cell cycle. Studies performed at our centre have shown the S-phase fraction of B-ALL to be characteristically around 10, whereas a fraction of more than 30, as seen in our patient, would be more typical of BLL.^6^ Comparisons could not be made with the other cases in the literature as S-phase fraction analysis was not reported. The Ki67 index detects a protein associated with cellular proliferation, which is present in all active stages of the cell cycle. Given that the Ki67 index and S-phase analysis measure different aspects of proliferation, these parameters are not always comparable. Notably, the Ki67 index is unhelpful in distinguishing B-ALL and BLL as values of over 95% can be anticipated in both.^6^ Despite the conflicting laboratory findings in our patient, the high S-phase fraction in the context of extranodal disease prompted the testing for, and confirmation of, the translocation t(8;14). B-cell lymphoma 2 (BCL2) expression is typically seen in acute lymphoblastic leukaemia and the possibility of a double-hit lymphoma was excluded by the presence of TdT.^2^ S-phase fraction analysis may therefore serve as an early indicator of this disease entity, albeit further investigation of its clinical utility is required. In summary, a combination of diagnostic modalities with evaluation by a multidisciplinary team is required to confirm this rare entity. We propose a laboratory approach to the diagnosis of lymphomas with features of B-ALL and BLL (Figure 4).

The therapeutic management of BLL differs significantly from B-ALL. The former requires high-intensity pulsed chemotherapy that is tailored to the high proliferative rate of the tumour. Furthermore, the duration of therapy is shorter as compared to the extended maintenance used in B-ALL.^7^ Our patient was instituted on a BLL treatment protocol, in line with the therapeutic approach described in the literature.^1,4,5^ There are however limited cases and minimal long-term follow-up data from which meaningful conclusions can be drawn. On a molecular level, there is a lack of consensus as to whether this entity represents a precursor B-cell at an intermediate stage of maturation and with aberrant MYC expression, or a mature B-cell with a less differentiated immunophenotype.

Furthermore, questions have been raised as to whether or not the presence of the translocation t(8;14) translates into an actual proliferation advantage. In an attempt to address these questions, one study analysed the molecular characteristics of neoplasms positive for IG-MYC rearrangement and with precursor B-cell phenotype in 12 patients. Aberrant variable diversity joining recombination was shown in five patients and activating N- and K-RAS mutations were detected in seven; all neoplasms had a DNA methylation profile that clustered with that of precursor B-cells. In light of these findings, the authors of the study posed the question as to whether or not cases with MYC rearrangement better fit a B-ALL profile.^9^ What is apparent from the literature is that further studies are required into the molecular behaviour of this entity to determine the prognostic implications and best therapy.

### Conclusion

Leukaemia or lymphoma with translocation t(8;14) or variant MYC translocation and precursor B-cell phenotype is a rare entity with varied clinical presentation. Awareness of this entity and a high index of suspicion are required by both clinicians (haematologists, oncologists) and pathologists to prevent a misdiagnosis. Further studies into the molecular and clinical behaviour of this tumour are required to optimise the therapeutic approach.
